# Split Reporter Systems in Viral Protein–Protein Interactions and Multimerization: Mechanisms and Applications

**DOI:** 10.3390/cells15100930

**Published:** 2026-05-19

**Authors:** Haseeb Ahmad, Faizan Masood, Uzair Iqbal, Mohamed Shaltout, Yunus Yukselten, Richard E. Sutton

**Affiliations:** Division of Infectious Diseases, Department of Medicine, Yale School of Medicine, New Haven, CT 06510, USA; haseeb.ahmad@yale.edu (H.A.); faizan.masood@yale.edu (F.M.); uzair.iqbal@yale.edu (U.I.); mohamed.shaltout@yale.edu (M.S.); yunus.yukselten@yale.edu (Y.Y.)

**Keywords:** Split reporter systems, protein-fragment complementation, viral protein–protein interactions, viral protein multimerization, luciferase complementation, NanoBiT, viral assembly, high-throughput screening, antiviral drug discovery

## Abstract

Protein–protein interactions (PPIs) are fundamental to viral replication, regulating processes such as assembly, genome packaging, and virion maturation. Despite their biological importance, these interactions remain challenging to study and are relatively underexploited as therapeutic targets. Split reporter systems, based on protein-fragment complementation, provide quantitative platforms to measure PPIs by reconstituting reporter activity when interacting protein partners are brought into proximity. These systems can be applied in vitro and in live cells which enables detection of dynamic and multimeric interactions in physiologically relevant contexts. Major classes of split reporter systems include β-lactamase, alkaline phosphatase, luciferase-based platforms, green fluorescent protein, and horseradish peroxidase. Assay performance depends on factors such as fusion protein stability, expression levels, and reporter kinetics, which influence sensitivity, dynamic range, and reliability. These approaches have been applied to study viral protein interactions across diverse systems, including HIV-1 matrix and nucleocapsid proteins, flaviviral capsid proteins, hepatitis B virus core protein, and chikungunya virus capsid. Split reporter assays also enable high-throughput screening for small-molecule inhibitors that disrupt viral PPIs and multimerization. This provides a functional readout linked to viral replication. Despite the challenges that exist in assay optimization and protein stability, the sensitivity and versatility of these systems provide a framework to interrogate viral protein interactions and support the development of antiviral therapeutics.:

## 1. Introduction

Protein–protein interactions (PPIs) are central to virtually all cellular processes, including signal transduction, structural organization, and macromolecular assembly. In viral systems, these interactions are particularly critical, as the coordinated multimerization of viral proteins drives essential steps such as genome encapsidation, capsid assembly, and virion maturation. Evidence suggests that disrupting these interactions can significantly impair viral replication, positioning PPIs as attractive but historically challenging targets for therapeutic intervention [1,2]. The transient and dynamic nature of many PPIs, combined with their dependence on cellular context, has limited the effectiveness of conventional biochemical methods in accurately capturing these interactions.

To overcome these challenges, protein-fragment complementation assays (PCA) have emerged as versatile and sensitive approaches for studying PPIs in living systems. These assays are based on the principle that a reporter protein can be split or divided into two inactive fragments that regain functionality when brought into proximity by two interacting protein partners. This strategy has been successfully applied to a range of reporter systems, including luciferases, fluorescent proteins, and enzyme-based reporters, enabling both qualitative and quantitative analysis of PPIs in real time [3,4]. Notably, luciferase-based complementation systems have gained widespread adoption due to their high sensitivity, low background signal, and enhanced dynamic range, making them particularly suitable for detecting weak or transient interactions in complex biological environments [5].

Beyond basic mechanistic studies, split reporter systems have become powerful tools in high-throughput screening (HTS) for the discovery of small-molecule inhibitors targeting PPIs. By coupling protein interaction events to a measurable signal output, these assays allow rapid and quantitative assessment of compound efficacy in physiologically relevant settings [6,7]. This approach is especially valuable in virology, where many viral proteins rely on oligomerization or multimerization for function and where traditional enzymatic targets are limited or prone to resistance. As a result, targeting protein multimerization has emerged as a promising strategy for antiviral drug development.

In this review, we aim to address several key questions regarding the use of divided or split reporter systems in virology. Specifically, how effectively do these systems capture viral protein–protein interactions and multimerization in physiologically relevant contexts? What are the relative strengths and limitations of different split reporter platforms in terms of sensitivity, dynamic range, and applicability? How have these approaches been applied to study viral assembly processes across diverse viruses? Finally, to what extent can split reporter systems be leveraged for the identification of small-molecule inhibitors targeting viral protein interactions? By addressing these questions, we seek to provide a structured framework for evaluating split reporter technologies and their role in advancing antiviral research. We further highlight their use in studying viral protein multimerization, focusing on representative systems such as HIV-1 structural proteins, flaviviral capsid proteins, hepatitis B virus core protein, and alphavirus capsid proteins.

## 2. Split β-Lactamase System

**Figure 1:**
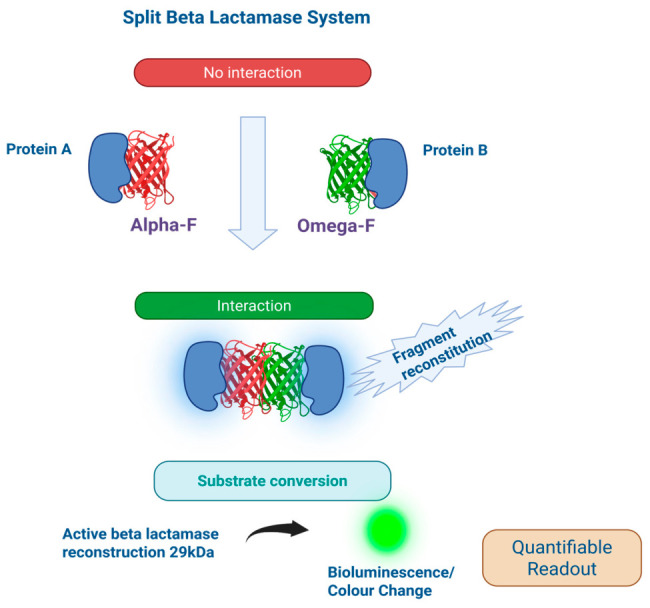
Split β-lactamase system. This is a protein-fragment complementation assay in which α and ω fragments reconstitute into an active enzyme upon protein–protein interaction. Part A: there is no interaction between proteins. Part B: protein interacts and beta lactamase components combine. Part C: the reassembled enzyme hydrolyzes β-lactam substrates, producing a measurable fluorescence or colorimetric signal that enables quantitative detection of molecular interactions.

The β-lactamase split system is a widely utilized protein-fragment complementation platform characterized by its modular design which typically comprises two inactive fragments (commonly referred to as α and ω) that reconstitute into an active enzyme upon molecular interaction, enabling flexible fusion to proteins of interest without significantly increasing construct size [8] [Figure 1]. The major strength of this system lies in its high sensitivity and signal amplification because enzymatic turnover of substrates produces robust and quantifiable readouts even at low levels of interaction [9,10]. For instance, split β-lactamase sensors have demonstrated greater than 40-fold specificity and femtomole-level detection sensitivity, highlighting their strong performance and broad dynamic range [9].

A notable limitation, however, is the presence of background activity due to spontaneous fragment reassembly or basal enzymatic activity, which can reduce the signal-to-noise ratio and affect assay precision [11,12]. Despite this, the β-lactamase split system maintains a favorable dynamic range, suitable for high-throughput and real-time applications, especially when optimized for substrate selection and expression conditions [9,12]. The utility of the β-lactamase split system is extensive. It spans applications in protein–protein interaction studies, ligand screening, live-cell signaling assays, and high-throughput drug discovery, with newer engineered variants further enhancing versatility through integration with protein splicing and intracellular detection platforms [8,13].

## 3. Split Alkaline Phosphatase System

**Figure 2:**
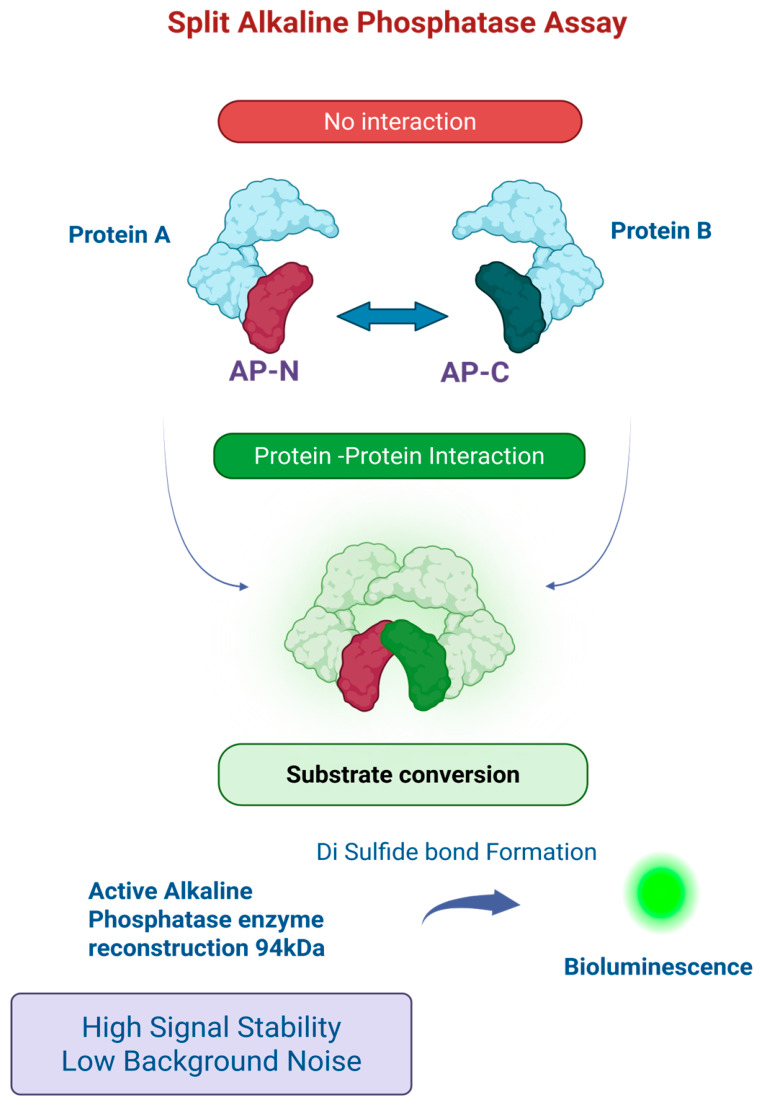
Split alkaline phosphatase system. This is a protein-fragment complementation assay. In first step inactive N- and C-terminal fragments reconstitute into an active enzyme upon protein–protein interaction. In second step the restored enzyme catalyzes substrate dephosphorylation, generating a detectable signal with high stability and low background. Shown is a bioluminescent signal, but it can be absorbance or fluorescence readout.

The split alkaline phosphatase (AP) system is a well-established protein-fragment complementation assay in which the enzyme is divided into two inactive fragments that regain catalytic activity upon proximity-driven reconstitution. It is typically derived from bacterial alkaline phosphatase (PhoA), and benefits from a relatively small and modular fragment design that facilitates fusion of interacting proteins with minimal steric hindrance [14] [Figure 2]. High signal stability and low background noise are its key strengths, as enzymatic activity is largely dependent on proper folding and disulfide bond formation, which reduces spontaneous reassembly [15,16]. Alkaline phosphatase exhibits strong catalytic turnover. This enables robust signal generation and makes it specifically suitable for applications requiring sustained signal output rather than rapid kinetics [8].

However, the requirement for an oxidizing environment to enable proper folding and disulfide bond formation represents a notable limitation of this system. This restricts its use primarily to periplasmic or extracellular environments, limiting intracellular applications [16,17]. When compared to other split reporter systems such as luciferases, the dynamic range of split AP may be moderate and require optimization of expression levels and substrate conditions to achieve maximal sensitivity [15,18]. Despite these limitations, the utility of the split AP system remains broad. Its applications include protein–protein interaction studies, membrane protein analysis, bacterial two-hybrid systems, and high-throughput screening platforms, where its stability and low background provide a reliable readout [14,17].

## 4. Split Gaussia Luciferase System

**Figure 3:**
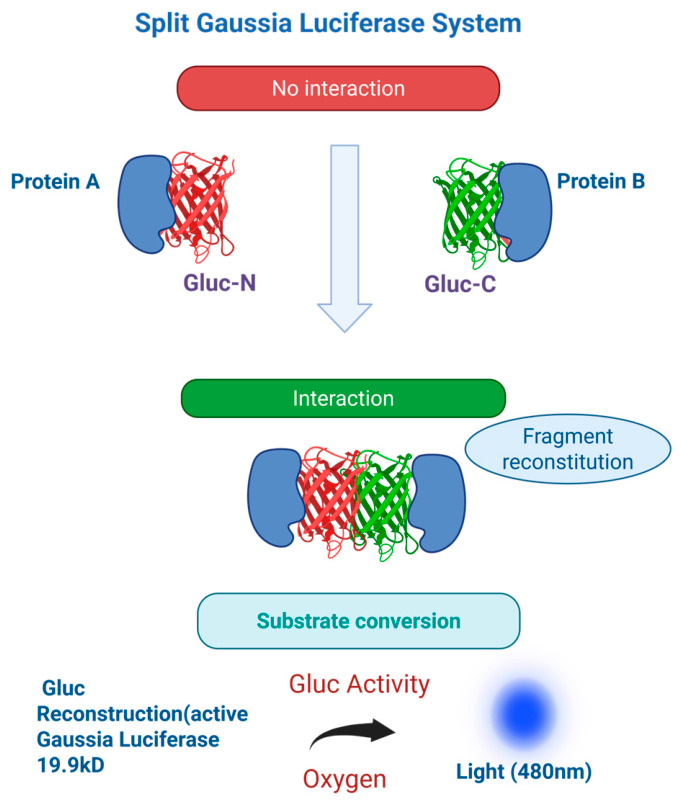
Schematic of the split Gaussia luciferase protein-fragment complementation assay. In first step, N- and C-terminal fragments of Gaussia luciferase are fused to proteins of interest (A and B). In second step, upon interaction the fragments reconstitute an active enzyme. In third step addition of coelenterazine substrate results in bioluminescence, enabling sensitive detection of protein–protein interactions.

The split luciferase system based on Gaussia luciferase (Gluc), is a highly sensitive protein-fragment complementation assay in which the enzyme is divided into two inactive fragments that reconstitute into an active bioluminescent enzyme upon molecular interaction. Gaussia luciferase is especially amenable to split design with minimal steric burden on fusion partners due to its naturally small size (~19.9 kDa) and strong light emission [19] [Figure 3]. One major strength of this system is its exceptionally high sensitivity and signal intensity, as bioluminescence-based detection offers near-zero background in mammalian cells compared to fluorescence-based reporters [20,21]. This results in a high edynamic range, enabling detection of weak or transient protein–protein interactions with good temporal resolution [20].

Limitations of the split Gaussia luciferase system include rapid signal decay due to flash-type kinetics and dependence on substrate (coelenterazine) stability, which can introduce variability in quantitative measurements [21]. Reconstitution efficiency may also vary depending on fusion orientation and expression levels, requiring empirical optimization [20]. Despite these challenges, the utility of split luciferase systems is extensive, including real-time monitoring of protein–protein interactions, in vivo imaging, drug screening, and signaling pathway analysis. The high sensitivity and low background makes it particularly advantageous for live cell and in vivo applications where subtle biological changes must be detected with precision [19,21].

## 5. Split Firefly Luciferase System

**Figure 4:**
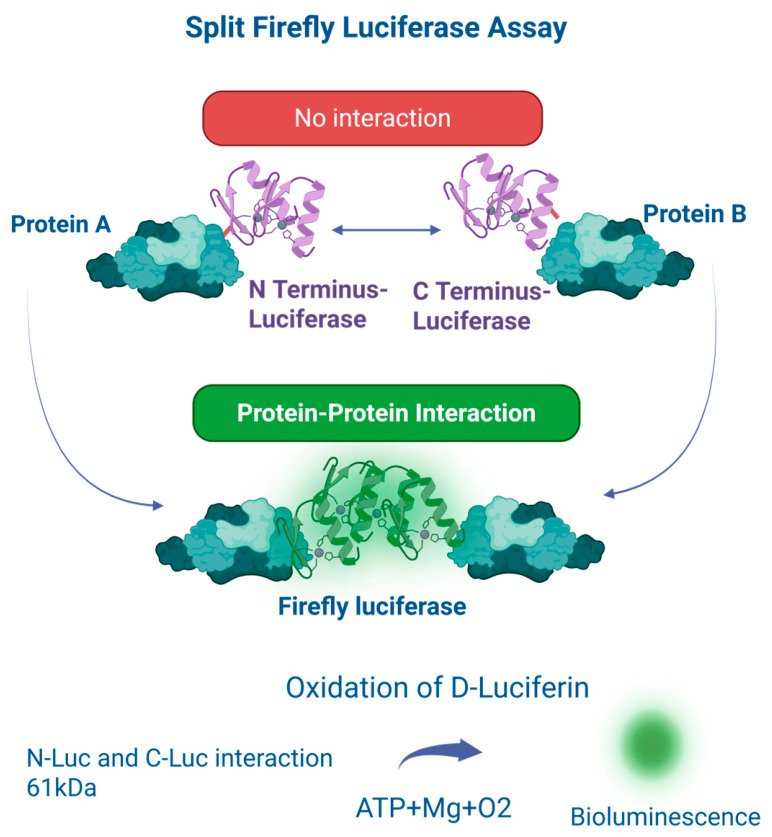
The split luciferase complementation assay. In first step Full-length FFLUC is split into two inactive fragments: the N-terminal (Nluc) and C-terminal (Cluc). In second step the Nluc is fused in-frame to protein A and Cluc is fused to protein B. When protein A binds to or interacts with protein B, it brings the two inactive enzyme fragments in close proximity, which results in a bioluminescent signal, after addition of ATP and luciferin.

Firefly luciferase (FFLuc) is derived from *Photinus pyralis.* It is a ~61 kDa bioluminescent enzyme widely used to study protein–protein interactions. It catalyzes the ATP-dependent oxidation of D-luciferin in the presence of oxygen and magnesium, producing visible light and enabling highly sensitive, quantitative detection [22]. In split luciferase complementation assays, FFLuc is divided into N- and C-terminal fragments fused to proteins of interest. Their interaction with each other restores enzymatic activity and generates a measurable luminescent signal [23,24] [Figure 4]. This approach allows direct, quantitative analysis of protein interactions in both cell-free and in cells. Split FFLuc offers high sensitivity, strong signal output, a broad dynamic range, and low background luminescence, resulting in high signal-to-background ratios suitable for quantitative and high-throughput applications [25].

Limitations, however, include its relatively large size, which may affect protein folding or function, and its dependence on ATP, which can introduce variability due to cellular metabolic state [23,25]. In addition, complementation is often partially irreversible, limiting detection of highly transient interactions [24]. Despite these constraints, split FFLuc assays are widely used in cell-based and in vitro systems to study protein interactions and signaling pathways. Its strong signal and low background makes it particularly suitable for high-throughput screening (HTS) to identify small-molecule inhibitors and to study protein multimerization [26]. Thus, split FFLUC assays remains a robust and versatile platform for mechanistic studies and large-scale screening.

A direct comparison between Gaussia and firefly luciferase-based split systems highlights important trade-offs relevant to experimental design. Gaussia luciferase offers higher intrinsic sensitivity and lower background due to its strong bioluminescent signal and small size, making it particularly suitable for detecting weak or transient protein–protein interactions. However, its flash-type kinetics and rapid signal decay can limit its utility in experiments requiring sustained or time-resolved measurements. In contrast, firefly luciferase produces a more stable glow-type signal, enabling more consistent quantitative measurements over time and making it well suited for high-throughput screening applications. This advantage comes at the cost of larger protein size and ATP dependence, which may introduce variability in certain cellular contexts. Therefore, the choice between these systems should be guided by the specific experimental requirements, particularly the need for sensitivity versus signal stability.

## 6. Split Green Fluorescent Protein (GFP) System

**Figure 5:**
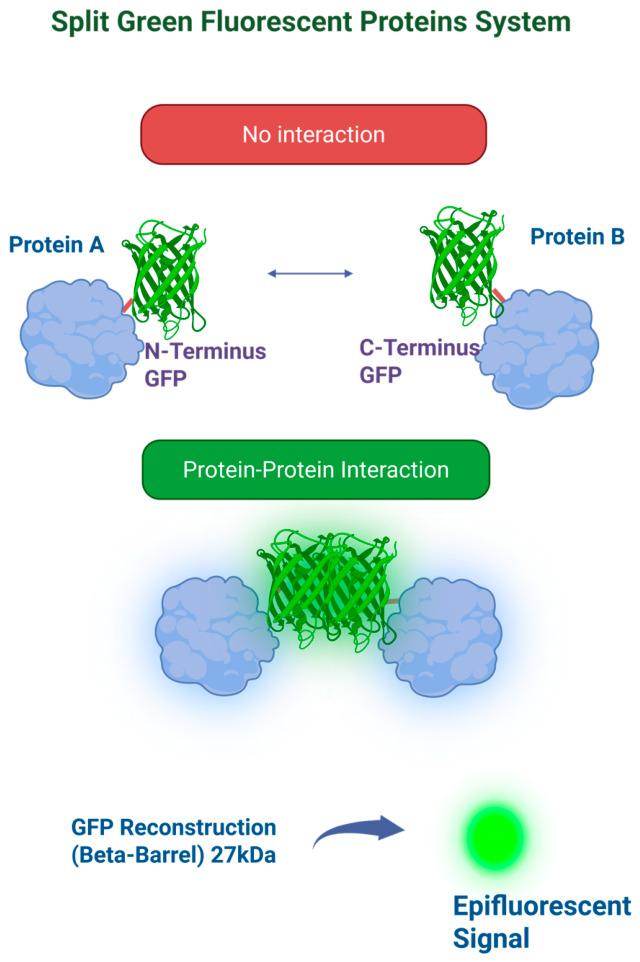
Split green fluorescent protein (GFP) system. This is a bimolecular fluorescence complementation system in which GFP is divided into two non-fluorescent fragments. In second step they reassemble upon protein–protein interaction. In third step this reconstitution restores the native GFP structure, resulting in a detectable epifluorescent signal that indicates that a molecular interaction occurred.

Split green fluorescent protein (GFP), used in bimolecular fluorescence complementation (BiFC) assays, is a fluorescence-based system for studying protein–protein interactions. GFP is derived from *Aequorea Victoria*, and it is a ~27 kDa protein that forms a stable β-barrel structure responsible for its intrinsic fluorescence [27]. In split GFP systems, the protein is divided into two non-fluorescent fragments fused to proteins of interest. The interaction between these proteins reconstitutes the native structure and restores fluorescence [4] [Figure 5]. A major advantage of this system is the ability to directly visualize protein interactions in living cells without exogenous substrates or cofactors, enabling spatial and temporal analysis using standard microscopy techniques [4,28]. Its relatively small size also reduces steric interference, improving compatibility with diverse fusion proteins [27].

However, split GFP has notable limitations. Complementation is mostly irreversible due to stable β-barrel formation, making it less suitable for studying transient interactions [4,28]. GFP maturation requires proper folding and chromophore formation, which can delay signal generation and reduce temporal resolution [27]. Its dynamic range and sensitivity are also lower than luminescence-based systems due to higher background and cellular autofluorescence [25]. For these reasons, split GFP is primarily used in cell-based assays for imaging and qualitative analysis of protein interactions, and is less suited for high-throughput screening (HTS) due to lower signal-to-background ratios and limited quantitative capability [4,25]. Despite these constraints, it remains a valuable tool for visualizing interaction localization in living cells, particularly in cell biology and virology.

## 7. Split Horseradish Peroxidase (HRP) System

**Figure 6:**
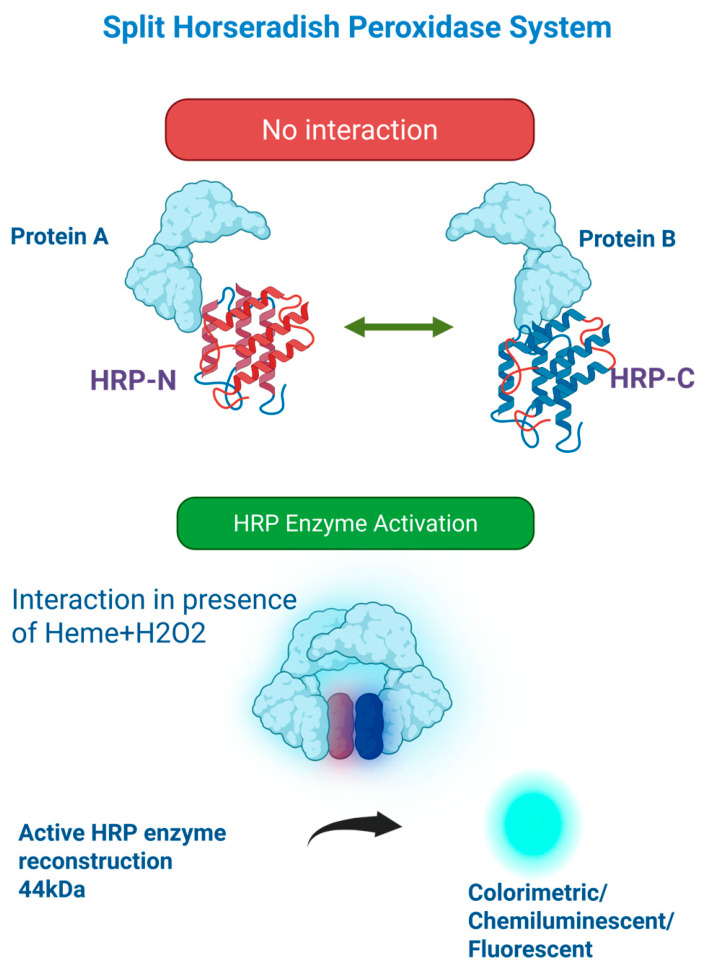
Split horseradish peroxidase (HRP) system. In first step the HRP has two terminals N and C attached to proteins. In second step two inactive fragments reconstitute into an active enzyme upon protein–protein interaction. In third step the reassembled HRP, in the presence of hydrogen peroxide and a heme cofactor, catalyzes substrate oxidation to generate detectable colorimetric, chemiluminescent, or fluorescent signals.

Split horseradish peroxidase (HRP) is an enzyme-based protein complementation system used to study protein–protein interactions, particularly in extracellular and membrane-associated environments. HRP is derived from *Armoracia rusticana* and it is a ~44 kDa heme-containing enzyme. It catalyzes substrate oxidation in the presence of hydrogen peroxide, producing colorimetric, chemiluminescent, or fluorescent signals [29]. In engineered systems, HRP fragments are fused to proteins of interest, enabling detection of protein interactions or proximity through reconstituted enzymatic activity [30] [Figure 6]. HRP provides signal amplification due to its catalytic activity. A single enzyme molecule can process multiple substrate molecules, resulting in high sensitivity [29]. It is well suited for studying interactions at the cell surface or within secretory pathways, where oxidizing conditions support enzyme activity, and it can be adapted to multiple detection formats for experimental flexibility [31].

However, HRP-based systems have their own limitations. Enzyme activity requires an oxidizing environment and heme incorporation, restricting use in reducing intracellular compartments such as the cytosol. This limitation arises from the requirement for oxidative folding, disulfide bond formation, and heme incorporation, processes that are largely restricted to the endoplasmic reticulum and are incompatible with the reducing environment of the cytosol. As a result, functional reconstitution of split HRP in intracellular compartments is inefficient, making it poorly suited for real-time monitoring of cytosolic protein–protein interactions in live cells. Background signal from endogenous peroxidases or non-specific substrate oxidation can reduce specificity. Assay performance, moreover, depends on substrate choice and reaction conditions, requiring careful optimization for reproducibility [29,31]. Although enzymatic amplification enhances signal, overall dynamic range is moderate and influenced by reaction kinetics. Split HRP is mainly used in cell-based systems for extracellular or membrane protein interactions, with limited application in cell-free systems. Although it is adaptable for high-throughput screening (HTS), it is less commonly used than luciferase-based assays due to higher background and more complex assay conditions [30,31]. Overall, split HRP is a useful platform in specific biological contexts, particularly at the cell surface, but its intracellular and HTS limitations have led to broader use of alternative systems.

Table below lists these various split reporter systems and the strengths and weaknesses of each [Table 1].

## 8. NanoLuc-Based Complementation Systems

Luciferase-based systems represent one of the most widely used and versatile platforms for studying protein–protein interactions in live cells due to their high sensitivity, low background, and compatibility with real-time kinetic measurements. Among these, split luciferase complementation systems, particularly NanoLuc-based NanoBiT, have substantially advanced intracellular protein interaction studies. NanoLuc is a small (~19 kDa) engineered luciferase derived from Oplophorus gracilirostris that exhibits high brightness, stability, and ATP-independent activity. In the NanoBiT system, NanoLuc is rationally split into a large fragment (LgBiT) and a small peptide fragment (SmBiT), which have low intrinsic affinity but efficiently reconstitute an active enzyme upon protein–protein interaction, producing a strong luminescent signal with minimal spontaneous association. This design enables highly sensitive detection of transient and low-affinity interactions, while the minimal size of SmBiT reduces steric interference and preserves native protein function. Consequently, NanoBiT has emerged as a benchmark system for real-time intracellular protein–protein interaction analysis in cytosolic, nuclear, and membrane-associated compartments [3].

In parallel, energy transfer-based approaches such as bioluminescence resonance energy transfer (BRET) and its optimized variant NanoBRET provide an orthogonal strategy for studying protein–protein interactions in living systems. These methods rely on non-radiative energy transfer from a luciferase donor to a fluorescent acceptor when interacting fusion partners are in close proximity, enabling quantitative measurement of interaction dynamics in real time. Classical BRET systems established the foundational framework for this approach by demonstrating its utility in monitoring protein interactions in live cells [32], while NanoBRET, which utilizes NanoLuc as the energy donor, improves brightness, spectral resolution, and signal-to-background ratio compared with earlier BRET configurations [33]. Although mechanistically distinct from split-protein complementation systems such as NanoBiT, BRET/NanoBRET approaches provide a complementary, distance-dependent method for validating protein interactions and interrogating their dynamic behavior under physiological conditions.

Despite their advantages, luciferase-based systems present distinct methodological considerations. NanoBiT assays depend on substrate availability and cellular uptake, which can introduce variability in signal intensity across experimental conditions, whereas BRET/NanoBRET systems require precise optimization of donor–acceptor geometry, spectral overlap, and expression ratios to ensure accurate energy transfer efficiency. Additionally, overly stable or irreversible complementation in split systems may limit the resolution of rapidly reversible interactions, while BRET-based systems may be more sensitive to structural constraints imposed by fusion design. Collectively, these luciferase-based platforms provide complementary and powerful approaches for dissecting protein–protein interactions, with NanoBiT offering high sensitivity and simplicity of readout, and BRET/NanoBRET enabling quantitative, distance-dependent interaction measurements in live-cell environments [3,32,33].

## 9. Application of Split Reporter Systems in Small-Molecule Inhibitor Screening

Protein-fragment complementation systems such as split β-lactamase, alkaline phosphatase, and luciferase are widely applied in the identification of small-molecule inhibitors by coupling enzyme reconstitution to biologically relevant molecular interactions. In these assays, the reporter enzyme is divided into two inactive fragments that are fused to interacting protein partners; upon interaction, enzymatic activity is restored, producing a measurable signal. Small-molecule inhibitors that disrupt the underlying protein–protein or protein–ligand interaction lead to a reduction in signal and this enables quantitative screening of inhibitory compounds in a high-throughput manner [24,34]. This approach is useful because it directly links molecular inhibition to a functional readout, allowing real-time and physiologically relevant assessment of compound activity in live cells [34]. Assay performance in these systems is commonly evaluated using the Z′ or Z factor, a statistical parameter that integrates signal dynamic range and variability to determine suitability for high-throughput screening, with values between 0.5 and 1.0 indicating robust and reliable assays (Equation (1)). An excellent signal to background or S/B is 5 or greater, whereas an outstanding one is 10–20.


(1) $$Z^{\prime }=1-\frac{3\left(\sigma_{p}-\sigma_{n}\right)}{\left|\mu_{p}-\mu_{n}\right|}$$

Z prime or Z′. This is a statistical parameter which measures assay quality, calculated from the means (μ) and standard deviations (σ) of positive (p) and negative (n) controls. Signal-to-background (S/B) is μ*_p_*/μ*_n_*.

Among these systems, split luciferase assays are especially favored for small molecule screening due to their high sensitivity, low background, and compatibility with both live-cell and in vivo formats. This enabled detection of subtle inhibitory effects with a high dynamic range [26,34]. Similarly, β-lactamase-based reporter assays have been extensively adapted for high throughput drug discovery platforms, including screening for inhibitors of viral replication and receptor signaling pathways, owing to their robust enzymatic amplification and compatibility with fluorescence-based substrates [5,27]. Although split alkaline phosphatase systems are less commonly used for intracellular inhibitor screening due to folding constraints, they remain valuable in membrane-associated and extracellular target studies where stable, low-background signal detection is required [14]. Collectively, these systems provide versatile and scalable platforms for small-molecule inhibitor discovery, with the choice of reporter determined by the required sensitivity, cellular context, and assay throughput [24,26,34].

## 10. HIV-1 Matrix Protein (MA) Multimerization

The human immunodeficiency virus type 1 (HIV-1) matrix (MA) protein is a structural component of the Gag polyprotein that plays a central role in viral assembly and budding. Following Gag cleavage, MA associates with the inner leaflet of the plasma membrane, where it directs viral particle assembly. The ~17 kDa protein is myristoylated at its N-terminus, a modification essential for its membrane binding and proper assembly [35,36]. MA multimerizes into higher-order oligomers and forms trimers that further organize into hexameric lattice-like structures inside the plasma membrane. This stabilizes assembly sites and coordinates the incorporation of viral components during virion formation [36,37].

MA multimerization is regulated by both protein–protein and protein–lipid interactions, particularly binding to phosphatidylinositol-(4,5)-bisphosphate [PI(4,5)P_2_], which enhances membrane targeting and promotes oligomerization [36,38]. Disruption of MA–MA interactions or membrane binding impairs viral assembly and reduces infectivity, underscoring its functional importance. Due to its well-defined structure and essential role, MA multimerization serves as a valuable model for studying protein–protein interactions. Structural studies have established its oligomeric organization [36,37]. Split reporter systems, including luciferase-based complementation assays, are widely used to monitor interactions and assess effects of mutations or small-molecule inhibitors [26]. Overall, MA represents an ideal system for investigating protein multimerization and for developing assays to identify inhibitors of viral assembly.

## 11. West Nile Virus Capsid Protein Multimerization

West Nile virus (WNV) is a member of the *Flaviviridae* family. It encodes a capsid (C) protein that plays a central role in viral genome packaging and nucleocapsid assembly. This small, highly basic ~12 kDa protein binds viral RNA and facilitates nucleocapsid core formation. It also participates in essential protein–protein interactions during virion assembly and maturation [39,40]. A defining feature of the WNV capsid protein is its ability to form homodimers, which serve as the fundamental structural unit. Structural studies show that the protein adopts an α-helical fold and dimerizes through hydrophobic interactions, forming a stable interface. These dimers can further assemble into higher-order oligomers that contribute to nucleocapsid organization, although their precise architecture remains less well defined [40,41].

Capsid multimerization is closely linked to interactions with viral RNA and host membranes. Its positively charged surface mediates electrostatic binding to the negatively charged viral genome. This promotes efficient encapsidation, while association with intracellular membranes such as the endoplasmic reticulum supports viral assembly [40,42]. Disruption of dimerization or RNA binding impairs particle formation and reduces infectivity, underscoring its functional importance. Due to its essential role, the WNV capsid protein serves as a useful model for studying protein–protein interactions. Structural studies have defined its dimeric organization [40,41], and split reporter systems, including luciferase- and fluorescence-based complementation assays, are widely used to monitor interactions and evaluate effects of mutations or small-molecule inhibitors. Its simple dimer-based organization and critical role in the viral life cycle make it a valuable model for investigating viral protein interactions and targeting viral assembly.

## 12. Flavivirus Capsid Protein Multimerization

West Nile virus (WNV) and Zika virus (ZIKV), both members of the Flaviviridae family, encode capsid (C) proteins that play central roles in viral genome packaging and nucleocapsid assembly. These small, highly basic ~12 kDa proteins bind viral RNA and facilitate nucleocapsid formation while also participating in protein–protein interactions required for virion assembly and maturation [39,43]. Similarly to other flaviviruses, capsid proteins of both WNV and ZIKV form homodimers that serve as the fundamental structural unit of the nucleocapsid. Structural studies demonstrate that these proteins adopt predominantly α-helical folds, with dimerization mediated largely through hydrophobic interactions [40,41,44]. These dimers can further assemble into higher-order oligomeric structures that contribute to nucleocapsid organization, although the precise architecture within mature virions remains incompletely defined.

Capsid multimerization in flaviviruses is closely linked to interactions with viral RNA and host cellular membranes. The positively charged surface of the capsid proteins mediates electrostatic binding to the negatively charged viral genome, promoting efficient encapsidation. In addition, interactions with intracellular membranes, including the endoplasmic reticulum and lipid droplets, support viral assembly and particle formation [40,42]. Disruption of capsid dimerization or RNA binding impairs particle assembly and reduces infectivity, underscoring the functional importance of these interactions.

Due to their relatively simple dimer-based organization and essential role in the viral life cycle, flaviviral capsid proteins serve as valuable models for studying viral protein–protein interactions and multimerization. Structural analyses have established the dimeric organization of both WNV and ZIKV capsid proteins [40,41,44], while split reporter systems, including luciferase- and fluorescence-based complementation assays, have been used to monitor capsid interactions and evaluate the effects of mutations or small-molecule inhibitors. These features make flavivirus capsid proteins attractive systems for investigating viral assembly mechanisms and for developing antiviral strategies targeting capsid multimerization.

## 13. HIV Nucleocapsid Multimerization

HIV-1 nucleocapsid (NC) protein is also derived from the Gag polyprotein. It plays a central role in viral genome packaging and assembly through its intrinsic ability to multimerize in the presence of nucleic acids. NC contains two highly conserved CCHC-type zinc finger motifs that mediate specific binding to viral RNA. This facilitates the condensation and stabilization of the genomic RNA into ribonucleoprotein complexes [45,46]. Multimerization of NC is largely driven by cooperative interactions between NC molecules and the viral RNA, where NC acts as a nucleic acid chaperone, promoting RNA dimerization, rearrangement, and proper folding required for efficient packaging into virions [46,47]. This RNA-mediated assembly enables the formation of higher-order NC complexes, which are critical for organizing the viral genome within the capsid structure.

The multimerization of NC is essential for multiple stages of the HIV replication cycle, including reverse transcription, genome packaging, and virion maturation. By facilitating RNA dimer stabilization and strand transfer during reverse transcription, NC ensures efficient synthesis of viral DNA and enhances replication fidelity [47,48]. Disruption of NC multimerization or its zinc finger integrity has been shown to impair viral infectivity, thus making it an attractive target for antiviral drug development [49]. Small molecules that interfere with NC-RNA interactions or zinc coordination can destabilize these multimeric complexes, thereby inhibiting proper genome packaging and viral maturation [49,50]. NC multimerization is not only structurally critical but also functionally indispensable for HIV replication. This underscores its importance as a therapeutic target.

## 14. Hepatitis B Virus Core Protein Multimerization

Hepatitis B virus (HBV) core protein (HBcAg) is a highly conserved structural protein. It plays a central role in nucleocapsid assembly through its intrinsic ability to self-assemble into multimeric structures. HBV core protein monomers rapidly form stable homodimers. The stable homodimers serve as the fundamental building blocks for higher-order assembly into icosahedral capsids which are typically composed of 180 or 240 subunits arranged with T = 3 or T = 4 symmetry [51,52]. This assembly process is driven by protein–protein interactions within the core domain and is further stabilized by interactions with pregenomic RNA (pgRNA) and viral polymerase [52]. The C-terminal domain of HBcAg is rich in arginine residues. It mediates nucleic acid binding and facilitates encapsidation of pgRNA, thereby linking multimerization directly to genome packaging [53].

Multimerization of the HBV core protein is essential for viral replication, as proper capsid assembly is required for reverse transcription of pgRNA into partially double-stranded DNA within the nucleocapsid [53,54]. Disruption of core protein assembly or capsid stability has been shown to inhibit viral replication. This highlights the importance of multimerization in the HBV life cycle [54]. The dynamic nature of capsid assembly allows for conformational flexibility required during genome maturation and virion secretion [55]. Small molecules known as core protein allosteric modulators (CpAMs) can either accelerate aberrant assembly or destabilize capsid formation, thereby impairing viral replication and representing a promising class of antiviral agents [55,56]. Consequently, HBV core protein multimerization is both structurally critical and therapeutically targetable, making it a key focus in antiviral drug development.

## 15. Chikungunya Virus Capsid Multimerization

Chikungunya (CHIKV) is an alphavirus within the *Togaviridae* family. CHIKV Capsid is a multifunctional structural protein that mediates viral RNA encapsidation and nucleocapsid assembly through its ability to multimerize. CHIKV capsid contains an N-terminal RNA-binding domain and a C-terminal serine protease domain, with the N-terminal region facilitating electrostatic interactions with the viral genomic RNA [57,58]. Capsid monomers assemble into higher-order oligomeric structures through cooperative protein–protein and protein–RNA interactions, leading to the formation of a stable nucleocapsid core [58]. This multimerization process is essential for condensing the viral RNA genome and organizing it into a compact structure suitable for incorporation into budding virions at the plasma membrane.

Multimerization of the CHIKV capsid is critical for efficient viral replication and infectivity. Proper nucleocapsid assembly ensures accurate genome packaging and coordination with envelope glycoproteins during virion budding [59]. Disruption of capsid oligomerization or capsid–RNA interactions has been shown to impair viral particle formation and significantly reduce infectivity, highlighting its essential role in the viral life cycle [59,60]. Capsid has also been reported to interact with host cellular pathways, including nuclear transport mechanisms, contributing to host shutoff and viral pathogenesis [60]. Given its central role in genome encapsidation and virion assembly, CHIKV capsid multimerization represents a potential target for antiviral strategies aimed at interfering with nucleocapsid formation and viral replication [59,60].

## 16. Conclusions

Protein–protein interactions and multimerization are essential to the assembly of most viruses, required for genome packaging and replication, making them attractive targets for therapeutic intervention. As outlined in this review, split reporter systems provide powerful and versatile tools for studying these interactions in physiologically relevant contexts. By enabling direct and sensitive detection of molecular interactions, these platforms overcome many limitations of traditional approaches.

Different reporter systems offer complementary advantages: enzyme-based reporters such as luciferases and β-lactamase provide high sensitivity and quantitative readouts suitable for high-throughput screening, whereas fluorescence-based systems such as split GFP allow visualization of interactions within living cells. Importantly, these technologies have been successfully applied to identify small-molecule inhibitors that disrupt critical viral protein interactions, highlighting their value in antiviral drug discovery.

While split reporter systems provide powerful tools for detecting protein–protein interactions and multimerization, they do not fully recapitulate the complexity of viral assembly and maturation. Viral assembly is a highly coordinated, multi-step process that involves not only protein–protein interactions but also protein–RNA interactions, membrane remodeling, host factor engagement, and dynamic spatial and temporal regulation within the cell. For example, higher-order lattice formation, membrane curvature during viral budding, and proteolytic maturation events such as Gag cleavage in HIV-1 are not directly captured by split reporter assays. Additionally, these systems typically measure proximity-based interactions and may not distinguish between functional assembly intermediates and non-productive associations. Therefore, while split reporter systems are highly valuable for dissecting specific interaction networks, they are best interpreted in conjunction with complementary structural, biochemical, and virological approaches to fully understand virion assembly and maturation.

Split-protein reporter systems serve as valuable complementary tools in drug discovery by enabling real-time, cell-based monitoring of protein–protein interactions. This role has supported target validation and optimization of therapeutically relevant compounds such as venetoclax. Continued advancements in reporter design and assay optimization are expected to further improve sensitivity, dynamic range, and applicability. Looking forward, emerging technologies are expected to further expand the capabilities and applications of split reporter systems in virology and antiviral discovery. Advances in next-generation luciferase platforms, improved fluorescent reporters, and engineered complementation systems are enhancing assay sensitivity, temporal resolution, and dynamic range. Integration of split reporter assays with high-throughput screening, live-cell imaging, structural biology, and artificial intelligence-assisted drug discovery may enable more comprehensive analysis of viral protein interaction networks and assembly pathways. In addition, the development of reversible and minimally perturbing reporter systems may improve the study of transient and dynamic interactions in physiologically relevant settings. Collectively, these advances are likely to strengthen the utility of split reporter technologies in both mechanistic virology research and the development of novel antiviral therapeutics. Overall, split reporter systems represent robust and adaptable platforms for dissecting protein interactions and accelerating the development of novel therapeutic strategies targeting viral assembly and replication.

## Figures and Tables

**Table 1:** Strengths and weaknesses of various split reporter systems.

**System**	**Strengths**	**Weaknesses**
β-lactamase	High sensitivity; ratiometric readout; live-cell compatible	Background from spontaneous complementation; requires substrate
Alkaline phosphatase	High stability; low background; sustained signal	Requires oxidizing conditions; limited intracellular use; slower kinetics
Gaussia luciferase	Very high sensitivity; low background; small size; wide dynamic range	Flash kinetics; substrate instability; signal variability
Firefly luciferase	Stable signal; high sensitivity; well-characterized	Larger size; ATP-dependent; moderate background
GFP	No substrate; real-time imaging; good for localization	Low sensitivity; auto fluorescence; slow maturation
HRP	Strong amplification; high sensitivity	Not live-cell compatible; requires substrate; background possible

## Data Availability

No new data were created or analyzed in this study.
